# A Rare Case of a Primary Unilateral Low-Grade Paratesticular Leiomyosarcoma in a 2 Years Old Dog

**DOI:** 10.3389/fvets.2019.00083

**Published:** 2019-03-22

**Authors:** Carolina Balao da Silva, Luis Gómez Gordo, Jesús María Cuesta Gerveno, Cristina Ortega-Ferrusola, Patricia Martín-Muñoz, Francisco Javier Duque Carrasco, Carlos Parejo, Fernando Peña Vega

**Affiliations:** ^1^VALORIZA, Polytechnic Institute of Portalegre, Superior Agrarian School of Elvas, Elvas, Portugal; ^2^Pathological Anatomy Service, Veterinary Teaching Hospital, Caceres, Spain; ^3^Laboratory of Equine Reproduction and Equine Spermatology, Veterinary Teaching Hospital, Caceres, Spain; ^4^Internal Medicine Service, Veterinary Teaching Hospital, Caceres, Spain; ^5^Pathological Anatomy Service, Hospital Espírito Santo E.P.E., Évora, Portugal

**Keywords:** dog, oncology, paratesticular, leiomyosarcoma, urogenital

## Abstract

A 2 years old dog was brought to the clinic with complains of testicular enlargement. The tissue was diffusely affected as confirmed by ultrasonographic examination, being the right testicle atrophied and the right epididymis enlarged, with loss of echotexture and presence of several anechogenic areas. The situation required the excision of the referred testicle and epididymis. Final diagnose made by histopathological analysis was primary unilateral low-grade paratesticular leiomyosarcoma. Scarce bibliography is found on this matter, with several cases reported on human, and none in dog. This case report is therefore an important milestone on the area of small animal oncology directly related to the reproductive tissue.

## Background

Paratesticular sarcomas are mesenchymal tumors that can be originated in soft tissues, such as the spermatic chord, inguinal canal, testicular tunic or epididymis (1). In humans, the incidence of soft tissue sarcoma has been reported to be 1.8–5.0 per 100,000 per year, with 1.5% affecting the male genital tract (2). Among paratesticular sarcomas, it has been reported in humans that the most common histological subtypes are liposarcoma, leiomyosarcoma, rhabdomyosarcoma, undifferentiated pleomorphic sarcoma and fibrosarcoma (3–5). The prognosis is usually considered poor, since recurrence and metastasis are common (6). Moreover, the mechanism and outcome of regional lymph node resection, radiotherapy and chemotherapy is reportedly unclear in the human species (6). In dogs, tumors of the testicular region are relatively common, representing more than 90% of all canine genital tract tumors (7). However, it rarely affects paratesticular structures, such as the spermatic cord, inguinal canal, testicular tunic or epididymis (1).

Different histological techniques are used to pathologic diagnosis. However, sometimes the tissue line is not obvious and other techniques are required. Indeed, immunohistochemistry (IHC) plays an important role in the diagnosis of soft tissue tumors (8, 9).

## Case presentation

**Figure 1:**
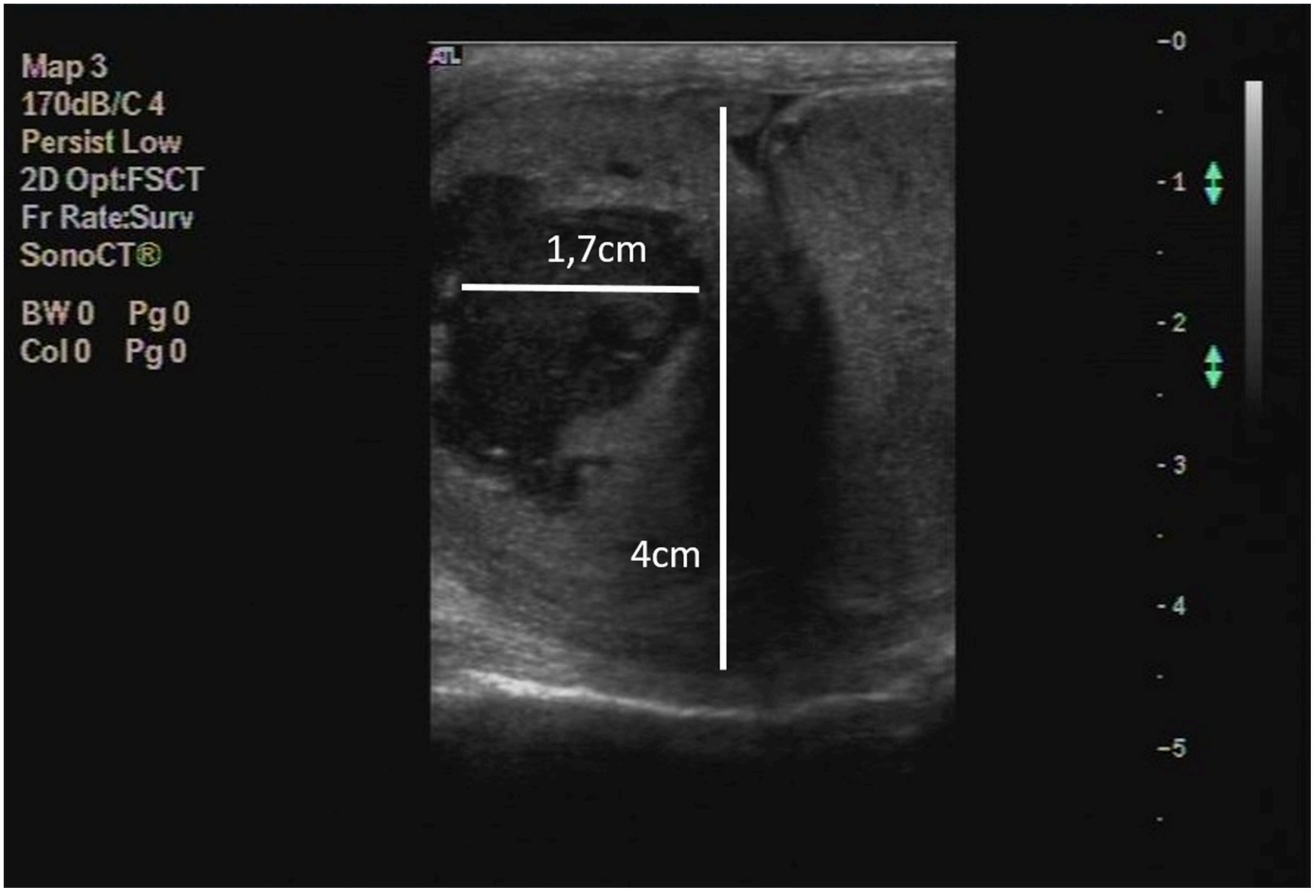
Testicle and epididymis, dog. Diagnostic ultrasonography of the epididymis (4 cm) with indication of one of the anechogenic areas (1.7 cm).

A 2 years old Spanish Mastiff was seen for the first time at the Veterinary Teaching Hospital of the University of Extremadura on April 2013. The complaint was scrotal enlargement starting ~3 weeks before. On scrotal palpation it was detected an apparently painless enlarged firm mass inside the right hemiscrotum with slightly increased temperature, when compared with the content of the left hemiscrotum. Physical examination also revealed parameters within normal limits, specifically temperature, mucosae, capillary retention, heart and respiratory rates, pulmonary auscultation and external lymph nodes. The size and consistency of the left testicle were apparently normal. A testicular ultrasonography was performed, major findings being: left testicle normal; right testicle atrophied; right epididymis presenting increased size (~4 cm), loss of normal echogenicity and presence of several anechogenic areas (<2 cm)(Figure 1).

A presumptive diagnosis of bacterial orchitis/epididymitis was proposed and treatment was started, with anti-inflammatory Meloxicam 24–24 h during 5 days (0.1 mg/kg; Metacam®, Boehringer Ingelheim, Ingelheim, Germany), and broad spectrum antibiotic Ciprofloxacin 12–12 h (20 mg/kg; Ciprofloxacino Normon, Madrid, Spain). The patient returned after 11 days and ultrasonography was repeated, presenting a reduction of the testicular size with persistence of the anechogenic areas. Due to the lack of regression of the lesions, surgical removal of the affected testis was proposed and unilateral orchiectomy was scheduled. Despite recommendation for bilateral castration, the owner decided to maintain the apparently healthy left testicle. Informed consent was signed by the owner respecting all the procedures reported, as well as the publication of this case report. Hematology and most biochemical parameters were within the normal range, except for total protein which presented a slightly reduced value (5 g/dL), with normal serum albumin (Tables 1, 2). Hemicastration of the right testicle was performed on the following week being the tissue sent for histopathology analysis. On the following 4 days, anti-inflammatory and antibiotic treatment was administered at the same doses as previously.

**Table 1:** Preoperative hematology.

**Parameter**	**Data of the animal**
Red blood cells (/mm^3^)	7,000,000
Hematocrit (%)	50.2
Hemoglobin (g/dL)	17.3
MCV (fl)	71.7
MCH (pg)	24.7
MCHC (g/dL)	34.5
White blood cells (/mm^3^)	10,340
Neutrophils	6,850
Eosinophils	770
Basophils	0
Lymphocytes	2,170
Monocytes	550
Platelet count	236,000

**Table 2:** Preoperative biochemistry.

**Parameter**	**Data of the animal**
Urea (mg/dL)	26.9
Creatinine (mg/dL)	0.8
ALT (UI/L)	19
Total bilirubin (mg/dL)	0.3
Total protein (g/dL)	5
Albumin (g/dL)	3.15

After 10 days the dog returned to follow-up, presenting increased inflammation on the area of excision, and presence of an anechogenical cavity on the interior. Inguinal lymph nodes were ultrasonographically normal, with no evidence of metastasis. Abdominal and thoracic radiographs were also made, with no gross abnormalities detected. Anti-inflammatory was again prescribed for 5 days, and antibiotic treatment was maintained for additional 15 days, maintaining previously used doses.

The excised testicle and epididymis were fixed in 5 per cent formaldehyde in a 0.1 M phosphate buffer (pH 7.2), processed and embedded in paraffin. For histopathological analysis, 5 μm sections were stained with hematoxylin-eosin and Masson Trichrome.

Histologically, the testis presented an extensive destruction of seminiferous tubules with a certain degree of intertubular connective tissue proliferation. Spermatogenic cells showed evident signs of degeneration, ranging from cellular swelling to apoptosis. The testicular parenchyma presented multiple inflammatory foci, consisting mainly of monocytes, lymphocytes and lesser degree plasma cells.

**Figure 2:**
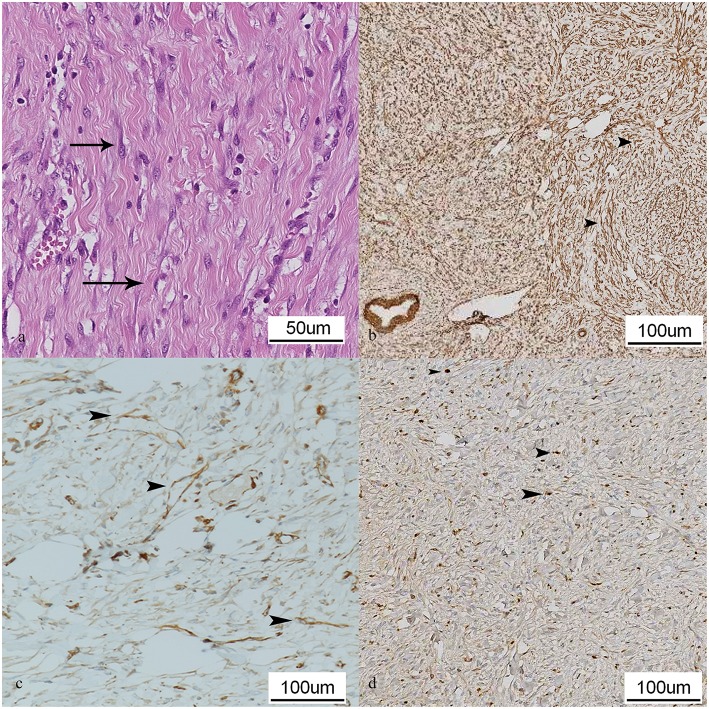
Paratesticular tissue, dog. **(a)** Histopathological study of the sample. Proliferation of loose connective tissue composed of fusocellular cells with slight atypia (arrows). Hematoxylin and eosin (HE). **(b)** Actin immunohistochemical stain. This stain highlights the presence of numerous cells which show a fusiform morphology (arrowheads). **(c)** Ki67 immunohistochemical stain of the lesion. Presence of fusiform cells expressing this marker, indicating cell proliferation (arrowheads). **(d)** Vimentin immunohistochemical stain in which it is observed several immunolabeled cells disposed with diffuse pattern and morphology that points to a mesenchymal origin (arrowheads).

Surrounding the testis, an abundant proliferation of loose connective tissue was shown, formed by fusocellular cells with slight atypia, without necrosis and with few atypical mitosis (1/10 HPF) (Figure 2a). This cell proliferation extended to the epididymis, causing a compressive phenomenon in its structures, which leads to two specific pathological changes, the disappearance of ducts and the dilation of the remaining ones by an increase of pressure, changing its cylindrical pseudostratified epithelium to an epithelium simple plane. In this tissue there were inflammatory cellular accumulations similar to those presented in the testis, although in a much lower number, with sketches of granulomas with giant cells, compatible with chronic inflammatory processes.

In order to classify the lesions, immunohistochemical study was performed using an automated slide stainer (Benchmark Ultra, Ventana®). Sections were immunostained using vimentin, actin, desmin, CD34, AE1/AE3 and Ki67 primary polyclonal antibodies (Roche®) according manufacturer's recommendations (Table 3). Bound Antibodies were visualized using avidin-biotin-peroxidase complex (ABC) technique and diaminobenzidine. Immunohistochemical study of cell proliferation showed positivity to Actin (Figure 2b), Ki67 (5–10%) (Figure 2c) and Vimentin (Figure 2d), and negativity to Desmin, CD34 and AE1/AE3, which indicates a low-grade leiomyosarcoma, according to recent studies (8).

**Table 3:** Details of primary antibodies.

**Antibody**	**Clon**	**Retrieval treatment**	**Incubation**
Vimentin	V9	Cell conditioning solution CC1/32 min	16 min
Actin	1A4	CC1/8 min	104 min
Desmin	DE-R-11	CC1/40 min	16 min
CD34	QBEnd/10	CC1/32 min	16 min
CK AE1/AE3	PCK26	Protease 4 min + CC1/8 min	8 min
KI-67	30-9	CC1/32 min	16 min

After 6 months from the surgery, the dog was present for clinical examination, presenting no alterations in the scrotum or in the left testicle, as well as in other structures of the abdominal cavity, assessed by ultrasonography. The owner was asked to return annually but it was not possible to continue follow-up of the animal.

## Discussion

Paratesticular tumors are a rare condition in humans (1) and in dogs (7, 10). The proximity and communication between the structures in the testis and paratestis result in a variety of tumors and tumor-like structures that present a diagnostic challenge due to their morphologic overlap and rarity (11). This heterologous group of tumors can follow different clinical courses, being histopathological subtype and tumor grade the most important features to determine prognosis (12). In the present case, the low-grade sarcoma with muscle differentiation found could be expected to have a lower risk of metastization, as confirmed in human spermatic cord sarcomas (13).

A retrospective study made in 2016 evaluated the most common tumor diagnoses in dogs in Switzerland between 1995 and 2008, comprising 63,214 neoplasic lesions, with soft tissues occupying the second higher risk after mammary gland, although no reference to paratesticular tumors was made (14). Extratesticular tumors have been found in previously neutered dogs, in the spermatic cord and within the scrotal tunic, or even arising from the prescrotal incision site (10). However, these neoplasms consisted mainly on Sertoli or interstitial cell tumors, suggesting trauma, embryological ectopic testicular tissue, polyorchism or testicular tissue transplantation during surgery (10).

Primary malignant neoplasia of the epididymis has been considered a very rare condition in humans (15). Although more frequent in adults, epididymal tumors have been reported in young boys (15) which occurred similarly in the present case, since the veterinary patient was 2 years old large breed dog. Nevertheless, paratesticular sarcomas in humans have been reported to occur in all age groups, typically presenting as slow growing, painless, firm scrotal or growing masses, ranging in size from small, barely palpable lesions to very large masses (1). In the present case, the physical exam was also compatible with the final diagnose of paratesticular tumor. In dogs, mixed germ cell–stromal tumors account for 7% of testicular tumors. Other less common tumors include hemangioma, granulosa cell, sarcoma, embryonal carcinoma, gonadoblastoma and lymphoma (7). Papillary carcinoma of the epididymis has been described in dog, as well as mesenchymal tumors, such as fibroma/fibrosarcoma and leiomyoma/leiomyosarcoma (16), such as on the present case. The sparse bibliography found on this matter available and the small number of published cases provide few information on prognosis, which, to the author's knowledge, was apparently favorable.

## Data Availability

All datasets generated for this study are included in the manuscript and/or the supplementary files.

## Author Contributions

CBS and LGG contributed to the writing of the manuscript and literature review. LGG, JCG, and CP contributed to the histopathological study, interpretation and description of the imaging finding. CBS, CO-F, PM-M, and FPV were responsible for the surgery and follow-up of the case. FDC was responsible for the ultrasonography imaging.

### Conflict of Interest Statement

The authors declare that the research was conducted in the absence of any commercial or financial relationships that could be construed as a potential conflict of interest.
